# One-minute heart rate recovery and clinical outcomes in transthyretin cardiac amyloidosis: incremental model fit beyond conventional CPET indices

**DOI:** 10.1093/ehjopen/oeag181

**Published:** 2026-10-22

**Authors:** Antonio Kösters, Nikita Ermolaev, Mahshid Eslami, Leonhard Lichtblau, Lisa Seilinger, Stefanie Pahr, Mariusz Grzeda, Roza Badr Eslam

**Affiliations:** Division of Cardiology, Department of Internal Medicine II, Medical University of Vienna, Spitalgasse 23, Vienna 1090, Austria; Division of Cardiology, Department of Internal Medicine II, Medical University of Vienna, Spitalgasse 23, Vienna 1090, Austria; Division of Cardiology, Department of Internal Medicine II, Medical University of Vienna, Spitalgasse 23, Vienna 1090, Austria; Division of Cardiology, Department of Internal Medicine II, Medical University of Vienna, Spitalgasse 23, Vienna 1090, Austria; Division of Cardiology, Department of Internal Medicine II, Medical University of Vienna, Spitalgasse 23, Vienna 1090, Austria; Division of Cardiology, Department of Internal Medicine II, Medical University of Vienna, Spitalgasse 23, Vienna 1090, Austria; Department of Population Health Sciences, Bristol Medical School, University of Bristol, Oakfield House, Oakfield Grove, Bristol BS8 2BN, United Kingdom; Galen Research Ltd, Koba, 100 Barbirolli Square, Manchester M2 3BD, United Kingdom; Division of Cardiology, Department of Internal Medicine II, Medical University of Vienna, Spitalgasse 23, Vienna 1090, Austria; Department of Clinical Pharmacology, Medical University of Vienna, Spitalgasse 23, Vienna 1090, Austria

**Keywords:** Transthyretin cardiac amyloidosis, Heart rate recovery, Cardiopulmonary exercise testing, Risk stratification, Heart-failure hospitalization

## Abstract

**Aims:**

To assess the association of 1-min heart rate recovery (HRR1) with clinical outcomes and its incremental contribution to model fit beyond peak VO_2_ and ventilatory efficiency (VE/VCO_2_ slope) in transthyretin cardiac amyloidosis (ATTR-CM).

**Methods and results:**

Consecutive adults with confirmed ATTR-CM undergoing symptom-limited cardiopulmonary exercise testing (CPET) were included in this single-centre observational study. During standardized active recovery at 24 W, HRR1 was defined as peak heart rate minus heart rate exactly 60 s after exercise. Cox proportional-hazards models evaluated all-cause mortality and the composite of mortality or first heart-failure hospitalization. Principal models adjusted for age, sex, peak VO_2_ and VE/VCO_2_ slope; NT-proBNP and National Amyloidosis Centre (NAC) stage were examined separately. Of 161 baseline patients, 11 with unverifiable heart-rate measurements were excluded, and exact HRR1 was available in 110. Mortality analysis included 105 patients with 33 deaths; composite-outcome analysis included 107 patients with 51 events. Median HRR1 was 15.5 bpm. In conventional CPET-adjusted models, each 10-bpm greater HRR1 was associated with lower hazards of mortality [hazard ratio (HR) 0.65, 95% confidence interval (CI) 0.44–0.95; *P* = 0.028] and the composite outcome (HR 0.74, 95% CI 0.57–0.97; *P* = 0.029). Adding HRR1 improved model fit (likelihood-ratio *P* = 0.017 and *P* = 0.021, respectively); apparent C-indices increased from 0.770 to 0.795 for mortality and from 0.695 to 0.699 for the composite outcome. Associations were non-significant after separate adjustment for NT-proBNP or NAC stage.

**Conclusion:**

HRR1 remained associated with outcomes after CPET adjustment and improved model fit. Discrimination gains were modest; value beyond biomarker staging was unproven.

## Graphical Abstract

**Graphical Abstract:**
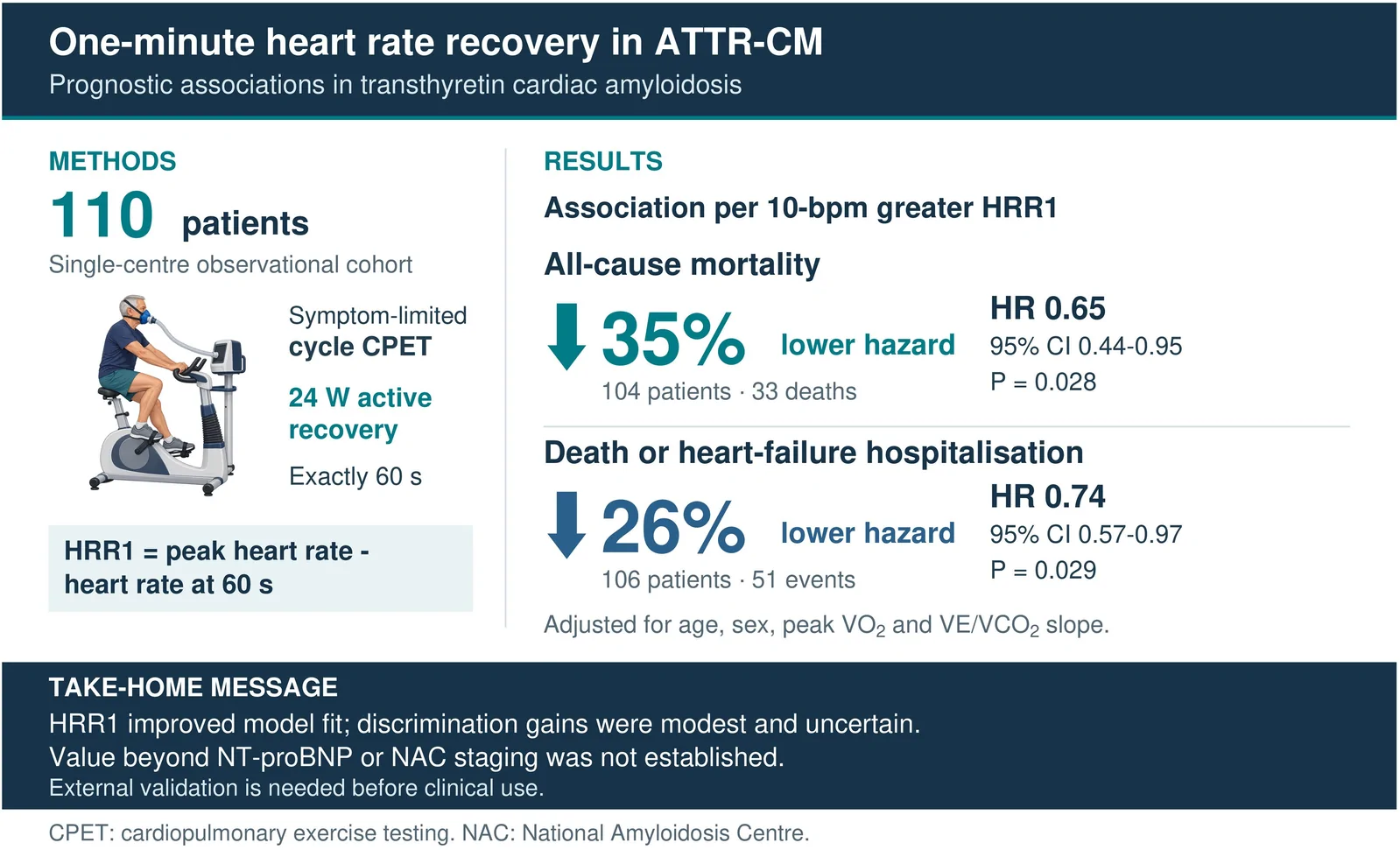

## Introduction

Transthyretin cardiac amyloidosis (ATTR-CM) is a progressive infiltrative cardiomyopathy caused by the deposition of misfolded wild-type or variant transthyretin fibrils within the myocardium. The resulting increase in myocardial stiffness and progressive impairment of cardiac function lead to heart failure, exercise intolerance, recurrent hospitalization, and premature mortality. Although tafamidis has been shown to reduce all-cause mortality and cardiovascular hospitalizations, the clinical course remains heterogeneous, emphasizing the need for accurate risk stratification.^1,2^ The National Amyloidosis Centre (NAC) staging system provides robust prognostic classification based on *n*-terminal pro-B-type natriuretic peptide (NT-proBNP) and estimated glomerular filtration rate.^1^ However, biomarker-based staging primarily reflects resting disease burden and may not fully characterize the integrated cardiovascular response to physical exertion.

Cardiopulmonary exercise testing (CPET) provides a comprehensive assessment of cardiovascular, ventilatory, and metabolic responses to exercise. Peak oxygen uptake (peak VO_2_) and the minute ventilation/carbon dioxide production (VE/VCO_2_) slope are the most established CPET-derived prognostic indices in heart failure. Previous investigations in cardiac amyloidosis have demonstrated that impaired peak VO_2_, reduced circulatory power, and increased VE/VCO_2_ slope are associated with mortality and heart-failure hospitalization.^3–6^ These findings support CPET for functional assessment and risk stratification in ATTR-CM. Nevertheless, existing studies have predominantly focused on variables obtained during exercise, whereas the potential prognostic relevance of the immediate post-exercise recovery phase remains insufficiently characterized.

One-minute heart rate recovery (HRR1), defined as the absolute decrease from peak heart rate to heart rate 1 min after exercise, is a simple marker of post-exercise cardiovascular recovery. The early decline in heart rate is predominantly mediated by parasympathetic reactivation and is blunted in patients with chronic heart failure.^7^ Delayed HRR1 has been associated with increased mortality across different cardiovascular populations.^8,9^ In patients with heart failure, HRR1 has also provided prognostic information beyond peak VO_2_ and VE/VCO_2_ slope, suggesting that it captures a physiological dimension not fully represented by conventional CPET indices.^9,10^ Abnormal chronotropic responses and impaired heart rate recovery appear to be common in ATTR-CM; in one prospective cohort, inadequate heart rate recovery was observed in 69% of patients.^11^ Although HRR1 has previously been associated with mortality in a broader ATTR cohort,^12^ its incremental value beyond peak VO_2_ and VE/VCO_2_ slope remains uncertain in ATTR-CM.

Accordingly, the present study investigated the association between HRR1 and clinical outcomes in patients with ATTR-CM undergoing CPET. We assessed all-cause mortality as the primary outcome and the composite of all-cause mortality or heart-failure hospitalization as the secondary outcome. We hypothesized that greater HRR1 would be associated with lower hazards and add prognostic information beyond age, sex, peak VO_2_ and VE/VCO_2_ slope.

## Methods

This was a single-centre observational cohort study of consecutive adults aged ≥18 years with confirmed ATTR-CM who underwent clinically indicated CPET at the Medical University of Vienna between June 2018 and February 2025. Data were obtained from prospectively maintained institutional cardiomyopathy and CPET registries. Patients with wild-type ATTR-CM and hereditary variant ATTR-CM were eligible.

ATTR-CM was diagnosed according to contemporary clinical criteria using an integrated assessment of clinical findings, cardiac imaging, laboratory investigations, bone-tracer scintigraphy, and histology where required. A non-biopsy diagnosis was permitted in patients with grade 2 or 3 myocardial uptake on bone-tracer scintigraphy and no evidence of a monoclonal gammopathy. Histological confirmation and amyloid typing were used when clinically indicated. Transthyretin genetic testing was performed to distinguish wild-type from variant ATTR-CM.^13^

Patients were required to have completed an interpretable, symptom-limited incremental CPET. Patients unable to complete an interpretable symptom-limited CPET or with a predominantly non-cardiac limitation to exercise, including severe pulmonary, orthopaedic, or neurological disease, were excluded.

The study was conducted in accordance with the Declaration of Helsinki and was approved by the Ethics Committee of the Medical University of Vienna (EK 1918/2019). All participants provided written informed consent.

## Cardiopulmonary exercise testing

Symptom-limited CPET was performed in the upright position on an electronically braked cycle ergometer using an individualized ramp protocol. Work-rate increments were selected to achieve an incremental exercise duration of ∼8–12 min. Respiratory gas exchange was measured breath-by-breath using a regularly calibrated metabolic system, in accordance with established CPET recommendations.^14^ Heart rate was monitored continuously by electrocardiography, and blood pressure was measured at regular intervals. Exercise was continued until volitional exhaustion, limiting symptoms, or a clinical indication for termination.

Peak oxygen uptake (peak VO_2_) was defined as the highest 30-second average oxygen uptake achieved during the final phase of exercise and was indexed to body weight in mL·kg^−1^·min^−1^. The minute ventilation/carbon dioxide production relationship (VE/VCO_2_ slope) was calculated by linear regression from rest to the respiratory compensation point. Peak heart rate was defined as the highest heart rate recorded during incremental exercise. Peak respiratory exchange ratio was recorded as an indicator of exercise effort.

## Heart rate recovery

Immediately after the end of incremental exercise, the ergometer workload was reduced to 24 W and patients continued pedalling during a standardized active recovery phase. HRR1 was calculated as:


$$\begin{array}{r} \mathrm{HRR}1=\mathrm{peak}\;\mathrm{exercise}\;\mathrm{heart}\;\mathrm{rate}\text{-}\mathrm{heart}\;\mathrm{rate}\;60\\ \mathrm{seconds}\;\mathrm{after}\;\mathrm{the}\;\mathrm{end}\;\mathrm{of}\;\mathrm{incremental}\;\mathrm{exercise}. \end{array}$$

A larger positive HRR1 therefore represented a more rapid reduction in heart rate during recovery. HRR1 was analysed primarily as a continuous variable, scaled per 10-bpm greater heart-rate reduction.

Only heart rates documented at exactly 60 s were included in the primary HRR1 analysis. Measurements obtained after shorter or longer recovery periods—for example, after 30, 40, or 90 s—were not extrapolated, rescaled, or pooled with exact 60-second measurements. Missing recovery values were not imputed. Examinations were excluded from HRR1 analyses when either the peak or 60-second recovery heart rate could not be reliably verified from the source CPET documentation.

As a secondary exploratory analysis, HRR1 was dichotomized at ≤12 beats per minute, based on the convention used in previous active-recovery studies.^8,15^ Because this threshold was derived using a different active treadmill cool-down protocol and has not been validated for 24-W cycle-ergometer recovery in ATTR-CM, continuous HRR1 was considered the primary exposure.

## Clinical assessment

Demographic characteristics, New York Heart Association functional class, comorbidities, medication, documented atrial fibrillation, and the presence of a rhythm device were obtained from the institutional registry and medical records. NT-proBNP and estimated glomerular filtration rate values obtained contemporaneously with the index CPET were recorded. Recorded AF denoted documented history/status and did not establish rhythm during CPET. Tafamidis initiation by the index CPET was derived from the first recorded treatment-start date; blank dates remained unknown. The diagnosis-to-CPET interval was calculated from the recorded registry diagnosis date and the index CPET date. Duration sensitivity analyses required a non-negative diagnosis-to-CPET interval; one patient whose CPET preceded the recorded diagnosis by 14 days was excluded only from these analyses. The tafamidis comparator comprised patients whose first recorded initiation occurred after CPET; a later start date did not identify a never-treated group, and initiation by CPET did not confirm ongoing treatment or adherence.

National Amyloidosis Centre (NAC) disease stage was determined using the established NT-proBNP and renal-function thresholds.^1^ Stage I was defined as NT-proBNP ≤3000 ng/L and estimated glomerular filtration rate ≥45 mL·min^−1^·1.73 m^−2^; stage III was defined as NT-proBNP >3000 ng/L and estimated glomerular filtration rate <45 mL·min^−1^·1.73 m^−2^; all remaining complete combinations were classified as stage II.

## Clinical outcomes and follow-up

The primary outcome was all-cause mortality. The secondary outcome was the composite of all-cause mortality or first heart-failure hospitalization for cardiac decompensation. Follow-up began on the date of the index CPET. For the composite outcome, the date of the first qualifying event was used. Cardiac decompensation events occurring before the index CPET were not counted as incident outcome events.

Outcome data were obtained from institutional medical records and clinical follow-up documentation. Patients without an outcome event were censored at the date of their last documented clinical contact. Analyses of mortality and the composite outcome were performed separately because the availability of valid follow-up differed between the two outcomes.

## Statistical analysis

Continuous variables were summarized as mean ± standard deviation when approximately normally distributed and as median with interquartile range otherwise. Categorical variables were presented as absolute numbers and percentages.

Time-to-event analyses used the index CPET as time zero and the outcome definitions and censoring rules described above.

The primary exposure was HRR1 analysed as a continuous variable. Hazard ratios were expressed per 10-bpm greater reduction in heart rate during the first minute of recovery. A hazard ratio below 1.0 therefore indicated a lower event rate with more rapid heart rate recovery. In a secondary exploratory analysis, HRR1 was dichotomized at ≤12 beats per minute. Because this threshold was derived using a different active-recovery protocol and has not been validated for 24-W cycle-ergometer recovery in ATTR-CM, continuous HRR1 was considered the primary exposure.

Associations between HRR1 and clinical outcomes were assessed using Cox proportional-hazards regression with Efron handling of tied event times. Covariates were selected *a priori* on clinical grounds without univariable screening or automated stepwise selection. Given the available number of outcome events, a limited hierarchical modelling strategy was used. The proportional-hazards assumption was assessed using scaled Schoenfeld residuals and formal covariate-by-log (follow-up time) interaction tests. The following models were evaluated separately:

1. An unadjusted model containing HRR1 alone;
2. A core model adjusted for age and sex;
3. A conventional CPET model adjusted for age, sex, peak VO_2_, and VE/VCO_2_ slope;
4. A Biomarker model adjusted for age, sex, and log_2_-transformed NT-proBNP; and
5. A disease-stage model adjusted for age, sex, and NAC stage.

NT-proBNP was log_2_-transformed because of its right-skewed distribution, such that a one-unit increase represented a doubling of NT-proBNP. The conventional CPET, NT-proBNP, and NAC-stage models were fitted separately to avoid constructing an over parameterized model relative to the number of available events. HRR1 and age were scaled per 10-unit increase, VE/VCO_2_ slope per five-unit increase, and peak VO_2_ per 1 mL·kg^−1^·min^−1^ when the effects of individual covariates were reported. NAC stage was entered as an ordinal variable coded 1–3.

The base CPET model containing age, sex, peak VO_2_ and VE/VCO_2_ slope was compared with an otherwise identical model adding HRR1. Nested models were compared using a likelihood-ratio test in the same complete-case population. Harrell’s concordance index was calculated for both models. The apparent change in C-index was reported with a paired percentile 95% confidence interval from 1000 successful subject-level bootstrap resamples, with both prespecified models fully refitted in each resample. The same sampled patients were used for both models, retaining all predictors.

Bootstrap internal validation estimated optimism-corrected C-indices, calibration slopes and prediction error.^16^ Optimism was estimated as bootstrap-sample performance minus performance of the bootstrap-fitted model in the original sample. The calibration slope was obtained by Cox regression of each model’s linear predictor over the full follow-up period; the ideal slope is 1. Absolute risk at 3 years (1095.75 days) was calculated from the Efron-consistent baseline cumulative hazard and compared with Kaplan–Meier estimates overall and by predicted-risk quartile. Three-year Brier scores used inverse-probability-of-censoring weights from the marginal Kaplan–Meier censoring distribution. Lower Brier scores indicate less overall prediction error. The interval for apparent ΔC-index was not interpreted as an interval for the optimism-corrected change.

Exploratory sensitivity analyses assessed the HRR1 threshold of ≤12 beats per minute and subgroups defined by recorded AF, beta-blocker therapy, implanted rhythm-device status, first recorded tafamidis initiation by CPET, NAC stage, diagnosis-to-CPET interval (<1 vs. ≥1 year), peak respiratory exchange ratio and ATTR genotype. Within-subgroup models were adjusted uniformly for age. Sex was omitted from these exploratory subgroup models because only seven women were included and several strata had no events among women; the principal models retained sex adjustment. Subgroup estimates were withheld when fewer than five events were available or estimation was unstable. This reporting floor does not ensure adequate precision. HRR1-by-variable interactions were tested by likelihood-ratio comparisons of otherwise identical CPET-adjusted models; NAC interaction testing compared stages I and II, and diagnosis interval was entered continuously in years. All sensitivity analyses were considered hypothesis-generating, without correction for multiple comparisons.

Additional pooled models added recorded AF, beta-blocker therapy, device implantation, calcium-channel blocker class, antiarrhythmic class, tafamidis initiation by CPET, diagnosis interval or relative recovery workload to the conventional CPET model, one variable at a time. Relative recovery workload was defined as 24 W divided by peak exercise workload. HRR1 estimates before and after adding each variable were compared in the identical complete-case sample. Analogous matched-sample comparisons were performed for the separate age- and sex-adjusted NT-proBNP and NAC-stage models.

Analyses were based on complete cases for HRR1, outcome follow-up, and all covariates included in the respective model. Missing values were not statistically imputed, and the numbers of patients and events were reported for each model. No formal sample-size calculation was performed because all eligible patients in the institutional cohort were considered. All statistical tests were two-sided, with *P* < 0.05 considered statistically significant. Marginal missingness and overlapping patterns were described, and baseline characteristics of the 110 patients included in the HRR1 cohort were compared with those of the 40 remaining data-quality patients without eligible exact HRR1 using standardized differences. Missingness was not assumed to be random.

All analyses were performed using Python version 3.12.14 (Python Software Foundation), with NumPy version 2.3.5 and SciPy version 1.17.0. Cox partial-likelihood estimation with Efron handling of ties, likelihood-ratio testing, Harrell’s concordance index, bootstrap internal validation, calibration assessment and proportional-hazards diagnostics were implemented in a reproducible Python analysis script.

## Results

The initial baseline cohort comprised 161 patients with ATTR-CM, including 46 deaths and 74 death or heart-failure hospitalization events. Eleven patients were excluded because their peak and/or recovery heart-rate measurements could not be reliably verified from the source CPET documentation. The remaining data-quality cohort therefore comprised 150 patients, among whom 40 deaths and 66 composite events occurred.

A source-verifiable peak heart rate and recovery heart rate recorded exactly 60 s after the end of incremental exercise were available in 110 patients. Their mean age was 78.6 ± 6.4 years, and 103 (93.6%) were male. Median HRR1 was 15.5 beats per minute [interquartile range (IQR) 9.0–25.8], with values ranging from 0 to 59 beats per minute. Forty-two patients (38.2%) had HRR1 ≤ 12 beats per minute. Median resting and peak heart rates were 74 bpm (IQR 67–85) and 123 bpm (IQR 108–141), respectively. Mean peak VO_2_ was 14.9 ± 4.3 mL·kg^−1^·min^−1^, median VE/VCO_2_ slope was 38.4 (IQR 34.0–43.9), and median peak respiratory exchange ratio was 1.10 (IQR 1.05–1.17). Recorded AF history was available in 96 patients, of whom 51 (53.1%) had AF; 14 patients had no recorded AF status. Additional baseline characteristics are presented in *Table 1*. Median HRR1 was 18.0 bpm (IQR 9.0–30.0) in patients with recorded AF and 15.0 bpm (IQR 9.0–24.0) in those without recorded AF (Mann–Whitney *P* = 0.454). Recorded AF type and HRR1 distributions are detailed in Supplementary material online, *Table S7*.

**Table 1:** Baseline characteristics of the 60-second HRR1 cohort

HRR1 cohort	
(*n* = 110)	
Demographic and clinical characteristics	
Age, years	78.6 ± 6.4
Male sex	103 (93.6)
ATTR-CM category: wild-type/variant	104 (94.5)/6 (5.5)
NYHA class I/II/III	23 (23)/53 (53)/24 (24)
Recorded AF history, *n*/*N* (%)	51/96 (53.1)
Permanent/paroxysmal/unspecified AF, *n*	30/14/7
AF history not recorded, *n*/*N* (%)	14/110 (12.7)
Beta-blocker therapy	63/109 (57.8)
Calcium-channel blocker use, *n*/*N* (%)	14/108 (13.0)
Any antiarrhythmic medication, *n*/*N* (%)	13/108 (12.0)
Tafamidis initiated by CPET, *n*/*N* (%)	41/98 (41.8)
Implanted rhythm device	15/103 (14.6)
Diagnosis-to-CPET interval, months	2.5 [1.4–8.5] (*n* = 86)
**Heart rate physiology**	
Resting heart rate, bpm	74 [67–85]
Peak heart rate, bpm	122.5 [108.2–140.5]
Peak heart rate, % predicted	86.5 [76–99.2]
HRR1, bpm	15.5 [9–25.8]
HRR1 ≤ 12 bpm	42 (38.2)
**Cardiopulmonary exercise testing**	
Peak VO_2_ (mL/min/kg)	14.9 ± 4.3
VE/VCO_2_ slope	38.4 [34–43.9]
Peak RER	1.1 [1.05–1.17]
**Biomarkers and disease stage**	
NT-proBNP, pg/mL	2335 [1214–3527]
eGFR, mL/min/1.73 m^2^	78.9 ± 11.5
NAC Stage I/II/III	56 (65.9)/28 (32.9)/1 (1.2)

Table 1 summarizes the demographic and clinical characteristics, heart-rate parameters, cardiopulmonary exercise testing variables, biomarkers, and disease stage of the exact 60-second HRR1 cohort (*n* = 110). Data are presented as mean ± standard deviation (SD) for normally distributed variables, median [interquartile range, IQR] for non-normally distributed variables, and *n* (%) or *n*/*N* (%) for categorical variables.

Abbreviations: AF, atrial fibrillation; ATTR-CM, transthyretin cardiac amyloidosis; bpm, beats per minute; CPET, cardiopulmonary exercise testing; eGFR, estimated glomerular filtration rate; HRR1, one-minute heart rate recovery; NAC, National Amyloidosis Centre; NT-proBNP, *n*-terminal pro-B-type natriuretic peptide; NYHA class, New York Heart Association functional class; RER, respiratory exchange ratio; VE/VCO_2_ slope, ventilation-to-carbon dioxide production slope; peak VO_2_, peak oxygen uptake.

Values are reported for patients with available data. Denominators may therefore differ between variables because of missing data. For categorical variables, percentages were calculated using the number of patients with available data for the respective variable as the denominator. The proportion with unrecorded AF history uses all 110 patients as the denominator; AF subtypes are reported as counts among patients with recorded AF. HRR1 was defined as peak heart rate minus heart rate exactly 60 s after the end of incremental exercise during active cycling recovery at 24 W.

Recorded AF refers to documented history/status and does not establish rhythm during CPET. Calcium-channel blocker and antiarrhythmic use are class-level variables; individual agents and doses were unavailable. Device status denotes implantation and does not establish pacemaker dependence.

Tafamidis initiation by CPET denotes a recorded first treatment-start date on or before the index CPET. Blank start dates were retained as unknown. Ongoing use, discontinuation and adherence could not be verified from start dates alone.

Diagnosis-to-CPET interval was calculated as the index CPET date minus the recorded diagnosis date, divided by 30.4375 to express months. Both dates were available in 86 patients after unique registry linkage. One CPET preceded the recorded diagnosis by 14 days; this signed interval was retained in the descriptive summary.

Valid mortality follow-up was available in 105 patients, of whom 33 died. The composite outcome analysis included 107 patients and 51 events. Using the reverse Kaplan–Meier method, median follow-up was 3.8 years for the mortality analysis and 5.6 years for the composite outcome analysis.

Within the 110-patient HRR1 cohort, AF status was unavailable in 14 patients, device status in seven, tafamidis start date in 12, NT-proBNP in 14 and NAC stage in 25. Diagnosis dates were missing in 24 patients, with one additional negative interval excluded from duration sensitivities. Beta-blocker status was missing in one patient and the VE/VCO_2_ slope in one. Sixty-two patients had all eight principal sensitivity and staging variables available; missingness patterns overlapped (see Supplementary material online, *Table S5*). Male representation was higher in the HRR1 cohort than among the 40 patients without eligible exact HRR1 (93.6% vs. 77.5%; standardized difference 0.47), supporting caution about selection bias (see Supplementary material online, *Table S6*).

## HRR1 and all-cause mortality

In unadjusted Cox regression, each 10-bpm greater HRR1 was associated with a lower risk of all-cause mortality [hazard ratio (HR) 0.67, 95% confidence interval (CI) 0.48–0.93; *P* = 0.015]. The association remained significant after adjustment for age and sex (HR 0.65, 95% CI 0.46–0.94; *P* = 0.020).

After additional adjustment for peak VO_2_ and VE/VCO_2_ slope, each 10-bpm greater HRR1 remained associated with an ∼35% lower mortality hazard (HR 0.65, 95% CI 0.44–0.95; *P* = 0.028; *n* = 104, 33 deaths). Within the same model, higher peak VO_2_ was associated with a lower mortality hazard (HR per 1 mL·kg^−1^·min^−1^ 0.85, 95% CI 0.76–0.96; *P* = 0.007), while a higher VE/VCO_2_ slope was associated with a greater mortality hazard (HR per five-unit increase 1.47, 95% CI 1.11–1.94; *P* = 0.006) (*Table 2* and *Figure 1*).

**Figure 1:**
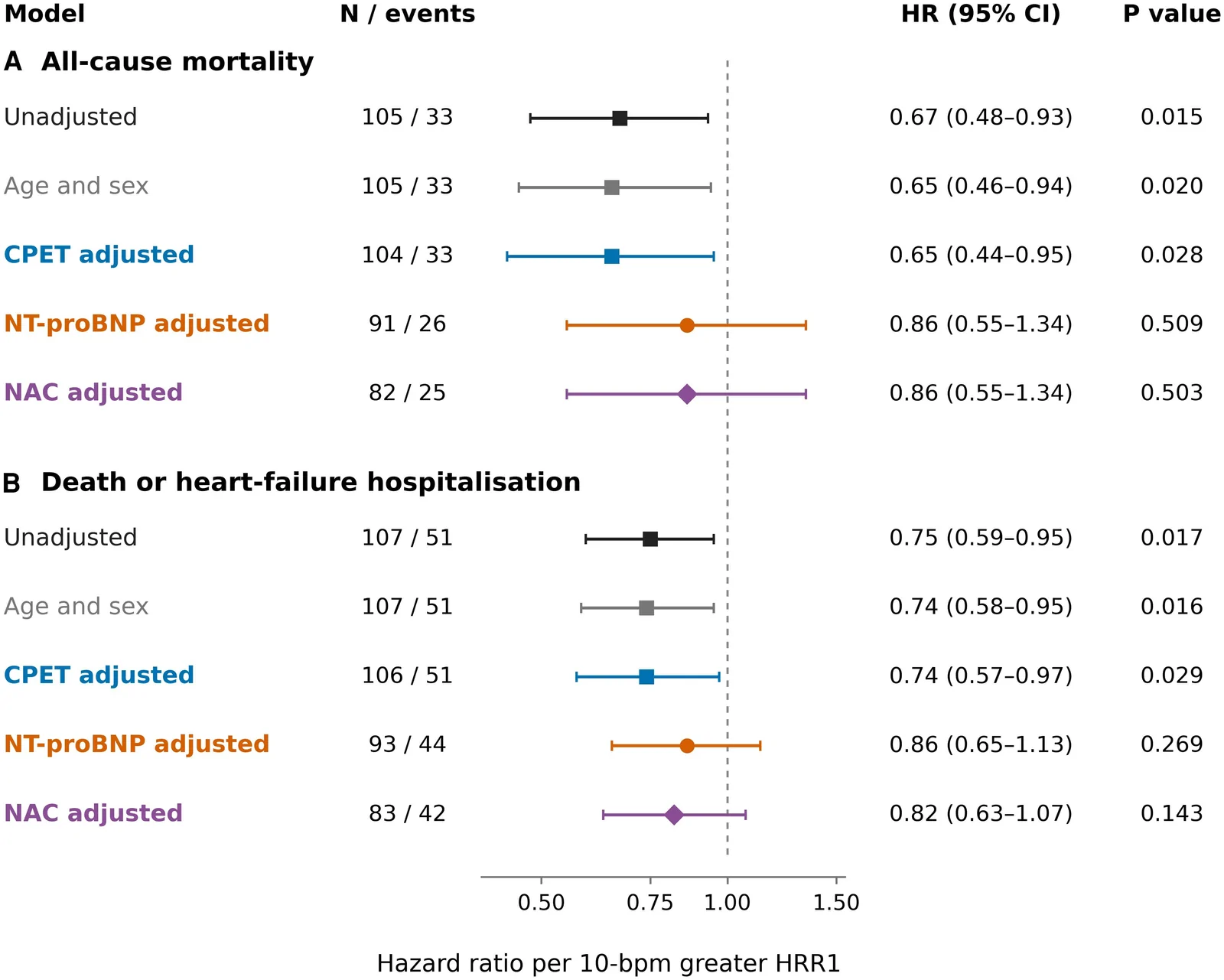
Association of continuous 1-min heart rate recovery with clinical outcomes. N/events denotes the number of patients and corresponding outcome events over follow-up in each model’s complete-case sample. Counts vary with follow-up and covariate availability. Markers indicate hazard ratios and horizontal lines indicate 95% confidence intervals per 10-bpm greater HRR1. Blue squares denote CPET-adjusted models, orange circles denote NT-proBNP-adjusted models, and purple diamonds denote NAC-adjusted models. Values below 1 indicate a lower hazard with greater heart rate recovery. All adjusted models include age and sex. The CPET-adjusted model additionally includes peak VO_2_ and VE/VCO_2_ slope; the NT-proBNP-adjusted model includes log_2_ NT-proBNP; and the NAC-adjusted model includes NAC stage. These are separate models, not cumulative adjustments. CI, confidence interval; CPET, cardiopulmonary exercise testing; HF, heart failure; HR, hazard ratio; HRR1, one-minute heart rate recovery; NAC, National Amyloidosis Centre; NT-proBNP, N-terminal pro-B-type natriuretic peptide.

**Table 2:** Association of continuous HRR1 with mortality and the composite outcome

Model	*n*	Events	HR (95% CI)	*P* value
**A All-cause mortality**				
Unadjusted	105	33	0.67 (0.48–0.93)	0.015
Age and sex	105	33	0.65 (0.46–0.94)	0.020
CPET adjusted	104	33	0.65 (0.44–0.95)	0.028
NT-proBNP adjusted	91	26	0.86 (0.55–1.34)	0.509
NAC adjusted	82	25	0.86 (0.55–1.34)	0.503
**B Death or heart-failure hospitalization**				
Unadjusted	107	51	0.75 (0.59–0.95)	0.017
Age and sex	107	51	0.74 (0.58–0.95)	0.016
CPET adjusted	106	51	0.74 (0.57–0.97)	0.029
NT-proBNP adjusted	93	44	0.86 (0.65–1.13)	0.269
NAC adjusted	83	42	0.82 (0.63–1.07)	0.143

Table 2 summarizes the associations between continuous HRR1 and all-cause mortality or the composite outcome of all-cause death or heart-failure hospitalization. Hazard ratios are reported per 10-bpm increase in HRR1, with values below 1 indicating a lower hazard with greater heart rate recovery. *n* indicates the number of patients included in the respective model, and Events indicates the corresponding number of outcome events. All adjusted models include age and sex. The CPET-adjusted model additionally includes peak VO_2_ and VE/VCO_2_ slope; the NT-proBNP-adjusted model includes log_2_ NT-proBNP; and the NAC-adjusted model includes NAC stage. These are separate models, not cumulative adjustments.

Abbreviations: CI, confidence interval; CPET, cardiopulmonary exercise testing; HF, heart failure; HR, hazard ratio; HRR1, one-minute heart rate recovery; NAC, National Amyloidosis Centre; NT-proBNP, *n*-terminal pro-B-type natriuretic peptide; VE/VCO_2_ slope, ventilation-to-carbon dioxide production slope; peak VO_2_, peak oxygen uptake.

The association between HRR1 and mortality was attenuated in the disease-severity models. After adjustment for age, sex, and log_2_-transformed NT-proBNP, the HR for each 10-bpm greater HRR1 was 0.86 (95% CI 0.55–1.34; *P* = 0.509; *n* = 91, 26 deaths). Similarly, after adjustment for age, sex, and NAC stage, the corresponding HR was 0.86 (95% CI 0.55–1.34; *P* = 0.503; *n* = 82, 25 deaths).

## HRR1 and death or heart-failure hospitalization

In the unadjusted analysis, each 10-bpm greater HRR1 was associated with a lower risk of the composite outcome of death or heart-failure hospitalization (HR 0.75, 95% CI 0.59–0.95; *P* = 0.017). This association remained significant after adjustment for age and sex (HR 0.74, 95% CI 0.58–0.95; *P* = 0.016).

After adjustment for age, sex, peak VO_2_, and VE/VCO_2_ slope, each 10-bpm greater HRR1 was associated with an ∼26% lower hazard of the composite outcome (HR 0.74, 95% CI 0.57–0.97; *P* = 0.029; *n* = 106, 51 events). Higher peak VO_2_ (HR per 1 mL·kg^−1^·min^−1^ 0.88, 95% CI 0.80–0.97; *P* = 0.012) and lower VE/VCO_2_ slope (HR per five-unit increase 1.24, 95% CI 1.01–1.53; *P* = 0.041) were also associated with the composite outcome.

As observed for mortality, the association was attenuated following adjustment for established measures of ATTR-CM disease severity. After adjustment for age, sex, and log_2_-transformed NT-proBNP, the HR was 0.86 per 10-bpm greater HRR1 (95% CI 0.65–1.13; *P* = 0.269; *n* = 93, 44 events). After adjustment for age, sex, and NAC stage, the corresponding HR was 0.82 (95% CI 0.63–1.07; *P* = 0.143; *n* = 83, 42 events).

## Incremental model fit, discrimination and calibration

Adding HRR1 to the CPET model improved fit for all-cause mortality (likelihood-ratio χ^2^ = 5.68; *P* = 0.017). The apparent C-index increased from 0.770 to 0.795, corresponding to an absolute increase of 0.025 (paired bootstrap 95% CI −0.010 to 0.094; *Table 3*).

**Table 3:** Incremental model fit, discrimination and calibration after adding HRR1 to conventional CPET indices

A Apparent discrimination and model fit						
Outcome	N/events	Base C-index	+ HRR1 C-index	ΔC-index (95% CI)	LR χ^2^	LR *P* value
All-cause mortality	104/33	0.770	0.795	+0.025 (−0.010 to 0.094)	5.68	0.017
Death or HF hospitalization	106/51	0.695	0.699	+0.004 (−0.020 to 0.062)	5.35	0.021

B Bootstrap optimism-corrected performance				
Outcome	Model	C-index	Calibration slope	3-year Brier score
All-cause mortality	Base	0.754	0.762	0.166
All-cause mortality	+HRR1	0.770	0.736	0.148
Death or HF hospitalization	Base	0.673	0.814	0.228
Death or HF hospitalization	+HRR1	0.670	0.785	0.228

The base model included age, sex, peak VO_2_ and VE/VCO_2_ slope; the extended model added continuous HRR1. N/events denotes the same complete-case population used for each nested comparison. Panel A reports apparent C-indices and paired percentile 95% confidence intervals for their difference from 1000 successful subject-level bootstrap resamples, with both models refitted in every resample. The interval describes apparent ΔC-index, not the optimism-corrected change. Panel B reports bootstrap optimism-corrected point estimates. Calibration slope uses full follow-up (ideal 1); the IPCW Brier score uses 3 years (1095.75 days), with lower values indicating less overall prediction error. Observed 3-year Kaplan–Meier risks were 24.5% (95% CI 16.5–35.5) for mortality and 40.6% (31.4–51.3) for the composite outcome; 49 and 45 patients remained at risk, respectively. A significant LR test indicates improved model fit and does not establish improved discrimination or clinical utility.

CI, confidence interval; CPET, cardiopulmonary exercise testing; HF, heart failure; HRR1, one-minute heart rate recovery; IPCW, inverse probability of censoring weighting; LR, likelihood ratio.

The corresponding likelihood-ratio test for the composite outcome was also significant (χ^2^ = 5.35; *P* = 0.021). The apparent C-index changed only from 0.695 to 0.699 (ΔC-index = 0.004; paired bootstrap 95% CI −0.020 to 0.062). Both confidence intervals included zero. After optimism correction, the C-indices were 0.754 and 0.770 for mortality and 0.673 and 0.670 for the composite outcome, for the base and HRR1-extended models, respectively.

Observed three-year Kaplan–Meier risks were 24.5% for mortality and 40.6% for the composite outcome. Mean apparent predicted risks were 24.7% and 25.0% for mortality and 39.9% and 40.6% for the composite outcome, for the base and HRR1-extended models, respectively. Bootstrap-corrected calibration slopes were below 1 for both models and outcomes, indicating some overfitting. The corrected three-year Brier score decreased from 0.166 to 0.148 for mortality and was ∼0.228 for both composite models (*Table 3*). Grouped apparent calibration is shown in Supplementary material online, *Figure S1*.

## Threshold and sensitivity analyses

When HRR1 was dichotomized at ≤12 beats per minute, a low HRR1 was not significantly associated with mortality after adjustment for age and sex (HR 1.82, 95% CI 0.90–3.69; *P* = 0.094). The corresponding association with the composite outcome was also not statistically significant (HR 1.64, 95% CI 0.94–2.88; *P* = 0.083). These findings supported retention of HRR1 as a continuous variable.

In age-adjusted analyses among patients without an implanted rhythm device (mortality: *n* = 85, 30 deaths; composite: *n* = 86, 43 events), greater HRR1 was associated with lower mortality (HR 0.67, 95% CI 0.48–0.94; P = 0.022) and composite risk (HR 0.73, 95% CI 0.56–0.94; *P* = 0.015). Associations were also present with peak respiratory exchange ratio ≥1.05 (mortality: *n* = 83, 24 deaths; HR 0.62, 95% CI 0.41–0.95; *P* = 0.028; composite: *n* = 85, 40 events; HR 0.72, 95% CI 0.54–0.96; *P* = 0.027) and in wild-type ATTR-CM (mortality: *n* = 96, 28 deaths; HR 0.62, 95% CI 0.42–0.92; *P* = 0.018; composite: *n* = 97, 45 events; HR 0.68, 95% CI 0.52–0.90; *P* = 0.006). All requested subgroup estimates, including those that were non-significant or not estimable, are presented in Supplementary material online, *Table S1*.

After additional adjustment of the conventional CPET model for recorded AF, HRR1 remained associated with mortality (*n* = 91, 32 deaths; HR 0.65, 95% CI 0.43–0.96; *P* = 0.032) and the composite outcome (*n* = 94, 50 events; HR 0.74, 95% CI 0.57–0.97; *P* = 0.029). Within-AF subgroup estimates were imprecise and significance was not consistent across outcomes and strata (see Supplementary material online, *Table S1*). CPET-adjusted HRR1-by-AF interaction tests were non-significant (likelihood-ratio *P* = 0.278 for mortality and *P* = 0.174 for the composite outcome); wide confidence intervals prevent exclusion of clinically meaningful heterogeneity (see Supplementary material online, *Table S3*).

Associations with both outcomes also persisted after separate additional adjustment for beta-blocker therapy, device implantation, calcium-channel blocker class or antiarrhythmic class (see Supplementary material online, *Table S2*). In the smaller samples with recorded tafamidis initiation or diagnosis interval, HRR1 was already non-significant before adding either variable, and estimates changed little after adjustment. Other interaction tests were non-significant, with limited precision (see Supplementary material online, *Table S3*). Only three deaths occurred among patients with an implanted device and among those tested ≥1 year after diagnosis, precluding subgroup mortality estimates under the reporting criterion. The single NAC stage III patient did not permit estimation.

The recovery workload was a median 33.8% of peak workload (IQR 24.0–43.6%; range 13.3–96.0%). After additional adjustment for the recovery-to-peak workload ratio, the mortality association was less precise (*n* = 104, 33 deaths; HR 0.694, 95% CI 0.480–1.004; *P* = 0.053), while the composite association remained nominally significant (*n* = 106, 51 events; HR 0.765, 95% CI 0.587–0.998; *P* = 0.048). This exploratory adjustment does not establish comparability across recovery protocols.

There was no statistically significant evidence of non-proportionality for HRR1 in the principal conventional CPET-adjusted mortality or composite models [covariate-by-log (follow-up time) interaction *P* = 0.199 and *P* = 0.079, respectively].

## Discussion

In this single-centre ATTR-CM cohort, greater HRR1 following symptom-limited CPET was associated with lower risks of all-cause mortality and death or heart-failure hospitalization. After adjustment for age, sex, peak VO_2_ and the VE/VCO_2_ slope, each 10-bpm greater HRR1 was associated with a 35% lower hazard of death and a 26% lower hazard of the composite outcome. The apparent gain in discrimination was larger for mortality than for the composite outcome, but both bootstrap confidence intervals for the change in C-index included zero. Optimism correction reduced the mortality gain and showed essentially no gain for the composite outcome. The associations were maintained in sensitivity analyses excluding patients with implanted cardiac devices, restricting the cohort to tests with a respiratory exchange ratio ≥1.05, and restricting the analysis to patients with wild-type ATTR-CM. However, HRR1 was no longer statistically significant after adjustment for NT-proBNP or National Amyloidosis Centre stage. These findings do not establish prognostic independence of HRR1 from biomarker-based measures of disease severity. Additional adjustment for recorded AF, beta-blocker therapy or device implantation preserved the associations, but subgroup estimates were imprecise. Neither the subgroup analyses nor the non-significant interaction tests resolve potential confounding by dynamic rhythm, pacing dependence or drug dose.

Contemporary recommendations emphasize the integration of clinical status, biomarkers, imaging and functional assessment in patients with ATTR-CM.^17^ Peak VO_2_ and the VE/VCO_2_ slope are the most extensively investigated CPET-derived prognostic parameters in cardiac amyloidosis.^3–6^ Restrictive pulmonary physiology and abnormal cardiopulmonary responses to exercise have also been described in ATTR-CM,^18^ while a systematic review and meta-analysis confirmed that lower peak VO_2_ and a higher VE/VCO_2_ slope are associated with mortality in cardiac amyloidosis.^19^ More recent investigations have expanded this evidence. In a cohort of 506 patients across the ATTR disease spectrum, Patel *et al*.^12^ demonstrated a high prevalence of chronotropic incompetence, impaired post-exercise heart rate reserve and ventilatory inefficiency.^12^ Longitudinal studies have shown that peak VO_2_ and ventilatory efficiency change during disease progression and treatment,^20^ while a multicentre investigation found particularly pronounced ventilatory inefficiency and an earlier anaerobic threshold in patients with amyloid cardiomyopathy compared with matched patients with non-amyloid heart failure.^21^ A subsequent meta-analysis of 17 studies and 1505 patients confirmed pervasive ventilatory inefficiency in ATTR amyloidosis and highlighted heterogeneity related to amyloid subtype and exercise modality.^22^ Combined CPET and exercise echocardiography has additionally demonstrated that changes in functional capacity may reflect changes in exercise stroke volume and cardiac output during disease-modifying treatment,^23^ and CPET-derived exercise capacity has been associated with quantitative myocardial DPD uptake and cardiac biomarkers.^24^

The present findings should particularly be considered in relation to the study by Patel *et al*.,^12^ in which ‘heart rate reserve’ was defined identically to HRR1 in our study, namely as peak heart rate minus heart rate 1 min into recovery.^12^ That investigation demonstrated that post-exercise heart rate reserve is frequently impaired across ATTR phenotypes. However, HRR1 was not evaluated as the principal prognostic exposure in models specifically designed to test its incremental contribution beyond peak VO_2_ and the VE/VCO_2_ slope. Their final multivariable prognostic analysis retained peak VO_2_ and peak systolic blood pressure. The present study therefore does not provide the first measurement of HRR1 in ATTR amyloidosis. It extends those observations by studying an exclusively ATTR-CM cohort and testing HRR1’s contribution to conventional CPET models. The association after CPET adjustment suggests that recovery captures information beyond peak VO_2_ and ventilatory efficiency.

The attenuation of the HRR1 association after adjustment for NT-proBNP and NAC stage requires careful interpretation. NT-proBNP is a powerful marker of haemodynamic stress and disease severity, while NAC stage combines NT-proBNP and renal function to provide validated prognostic stratification in ATTR-CM.^1^ The strength of biomarker-based staging was recently reconfirmed in more than 2000 patients, in whom expansion of the NAC system with a fourth stage identified patients at particularly high risk of early mortality.^25^ The movement of the HRR1 effect estimates towards unity after adjustment for NT-proBNP or NAC stage suggests that HRR1 and biomarker-based staging capture partially overlapping aspects of disease severity. In the matched biomarker-available samples, the age- and sex-adjusted HRR1 associations were already non-significant before adding NT-proBNP or NAC stage, and estimates moved towards unity after adjustment (see Supplementary material online, *Table S4*). Cohort selection and reduced precision may therefore contribute to the attenuation alongside shared prognostic information. The present data cannot determine whether the attenuation resulted predominantly from biological overlap, reduced statistical power or a combination of both. Accordingly, the findings should not be interpreted as demonstrating that HRR1 outperforms peak VO_2_, the VE/VCO_2_ slope, NT-proBNP or NAC stage.

Several mechanisms may underlie the association between impaired HRR1 and adverse outcomes. The initial decline in heart rate after exercise is predominantly mediated by parasympathetic reactivation, accompanied by a progressively increasing contribution from sympathetic withdrawal.^7^ Impaired heart rate recovery has been associated with mortality in unselected exercise-testing populations and in patients with chronic heart failure.^8–10,15^ Quantitative cardiovascular reflex testing has also demonstrated autonomic dysfunction in symptomatic and presymptomatic hereditary ATTR amyloidosis,^26^ and longitudinal evidence suggests that cardiovascular autonomic failure may occur early and progress during hereditary ATTR disease.^27^ These observations provide biological plausibility for abnormal post-exercise heart rate regulation, although they derive mainly from hereditary ATTR populations and cannot be directly extrapolated to a predominantly wild-type ATTR-CM cohort.

Across broader cardiovascular populations, a meta-analysis of prospective cohorts found a graded association of slower heart rate recovery with cardiovascular events and all-cause mortality, including when analysed per 10-bpm decrement.^28^ Data from more than 40 000 UK Biobank participants also suggested that very early recovery, particularly during the first seconds after exercise cessation, may contain stronger prognostic information than later measurements.^29^ Nevertheless, HRR1 should not be interpreted as a pure or specific measure of parasympathetic function. In ATTR-CM, it may integrate autonomic regulation, chronotropic competence, haemodynamic reserve, right ventricular function, rhythm, medication effects and the ability to reduce cardiovascular demand following maximal exercise.

The active recovery protocol is particularly relevant when interpreting the present findings. Recovery was performed while cycling at a workload of 24 W, meaning that HRR1 reflects cardiovascular deceleration during continued cycling rather than during complete rest. Experimental evidence indicates that muscle mechanoreflex activation during recovery may influence the early decline in heart rate.^30^ Moreover, exercise modality contributes to variation in CPET measurements across cardiac amyloidosis studies.^22^ Although the conventional HRR1 threshold of ≤12 bpm was originally derived during active treadmill recovery,^8,15^ the exercise modality and recovery intensity differed substantially from the present protocol. A fixed workload of 24 W also represents different relative intensities according to an individual patient’s exercise capacity. The absence of statistically significant associations for the ≤12-bpm threshold should therefore not be regarded as contradicting the continuous analyses. Rather, it indicates that this conventional threshold cannot automatically be transferred to active cycle recovery at 24 W. Protocol-specific reference values and clinically meaningful thresholds will require prospective validation.

From a clinical perspective, HRR1 has several potentially attractive characteristics. It can be obtained from a standard CPET without additional equipment, testing time or complex post-processing and requires only the heart rate values recorded at peak exercise and exactly 60 s into recovery. Statistical significance in a likelihood-ratio test does not establish clinically useful risk classification. The bootstrap-corrected calibration slopes below 1 indicate overfitting, and the negligible corrected discrimination gain for the composite outcome limits claims of clinical utility. Before clinical implementation, HRR1 requires external validation under a standardized recovery protocol. Future studies should investigate its reproducibility, external calibration, optimal functional form and clinical net benefit, as well as its incremental contribution beyond both CPET variables and biomarker-based staging. Continuous beat-to-beat recovery kinetics may also provide more detailed information than a single 1-min measurement.

## Limitations

First, this was a single-centre observational study, which limits generalizability and precludes causal inference. Residual confounding, including by disease-modifying treatment and other unmeasured determinants of prognosis, cannot be excluded. The sample size and number of events were limited, particularly for the mortality analyses relative to the number of model covariates. Although the data-quality cohort included 150 patients, HRR1 measured at exactly 60 s was available in only 110. The principal mortality and composite analyses consequently included 105 patients with 33 deaths and 107 patients with 51 composite events, respectively. Analyses involving NT-proBNP and NAC stage included still fewer patients and events. Complete-case analysis may have introduced selection bias, while the limited number of events increases statistical uncertainty and the possibility of model overfitting. Bootstrap internal validation quantifies some of this optimism but cannot replace external validation. The predominance of men and wild-type ATTR-CM, together with the near absence of NAC stage III patients, limits generalizability to women, patients with variant ATTR-CM and those with advanced disease.

Second, HRR1 was measured during active recovery at a fixed workload of 24 W. The observed effect estimates may not be transferable to passive recovery, unloaded cycling or treadmill protocols. The fixed absolute recovery workload may also have represented markedly different relative intensities between patients. Furthermore, only the 1-min recovery value was evaluated. Continuous recovery kinetics or later recovery measurements might provide a more comprehensive assessment. Adjustment for the recovery-to-peak workload ratio made the mortality association less precise and cannot remove the physiological effects of continued cycling or define a standard relative recovery intensity.

Third, cardiac rhythm throughout exercise and recovery was not manually adjudicated from the complete CPET tracing in every patient. Atrial fibrillation, ventricular pacing, conduction disease, chronotropic incompetence and rate-modifying medication may influence both peak heart rate and subsequent recovery. Recorded AF history, beta-blocker treatment and device implantation cannot establish rhythm during CPET or pacing dependence, or fully capture these dynamic effects. Beta-blocker dose, amiodarone-specific use, pacemaker dependence and ECG-defined baseline CPET rhythm were unavailable in the structured dataset. The added sensitivity analyses use these recorded baseline characteristics and cannot substitute for adjudication of the exercise and recovery tracings. Sparse events within several strata and the limited number of women also restrict assessment of effect modification.

## Conclusion

In patients with ATTR-CM, greater HRR1 during active recovery was associated with lower hazards of all-cause mortality and the composite of death or heart-failure hospitalization. The associations persisted after CPET adjustment, and adding HRR1 improved fit. Gains in discrimination were modest and uncertain after internal validation, and the associations were attenuated after adjustment for NT-proBNP or NAC stage. The value of HRR1 beyond biomarker staging and its clinical applicability require prospective external validation.

## Supplementary Material

oeag181_Supplementary_Data

## Data Availability

Data will be shared upon reasonable request to the corresponding author.
