# Supplementary material for: One-minute heart rate recovery and clinical outcomes in transthyretin cardiac amyloidosis: incremental model fit beyond conventional CPET indices
Source: Eur Heart J Open. 2026 Oct 22;6(6):oeag181. doi: 10.1093/ehjopen/oeag181 (PMC13728343; doi:10.1093/ehjopen/oeag181)
Supplement: Supplementary file 1 [file oeag181_supplementary_data.docx]

HRR1 supplementary material

Supplementary Tables S1–S7 and Figure S1 accompany the study of one-minute heart rate recovery in ATTR-CM. Sensitivity analyses use the 110-patient HRR1 cohort, with model-specific complete cases. HRR1 estimates are per 10-bpm greater recovery; exploratory analyses do not establish rhythm-specific or biomarker-independent prognostic value.

# Supplementary Table S1 Age adjusted subgroup analyses

| **Variable** | **Stratum** | **Death n/events** | **HR (95% CI)** | **P** | **Composite n/events** | **HR (95% CI)** | **P** |
| --- | --- | --- | --- | --- | --- | --- | --- |
| AF | No recorded AF | 42/12 | 0.36 (0.16–0.82) | 0.015 | 45/22 | 0.79 (0.55–1.13) | 0.200 |
| AF | Any recorded AF | 50/20 | 0.78 (0.54–1.12) | 0.171 | 50/28 | 0.68 (0.49–0.95) | 0.022 |
| Beta-blocker | No beta-blocker | 44/11 | 0.58 (0.30–1.12) | 0.105 | 43/17 | 0.51 (0.31–0.85) | 0.010 |
| Beta-blocker | Beta-blocker | 60/22 | 0.70 (0.47–1.03) | 0.073 | 63/34 | 0.88 (0.67–1.15) | 0.346 |
| Device | No device | 85/30 | 0.67 (0.48–0.94) | 0.022 | 86/43 | 0.73 (0.56–0.94) | 0.015 |
| Device | Device implanted | 14/3 | NE | NE | 14/7 | 0.55 (0.23–1.32) | 0.178 |
| Tafamidis start | Before or at CPET | 38/11 | 0.61 (0.32–1.16) | 0.131 | 39/18 | 0.95 (0.64–1.43) | 0.822 |
| Tafamidis start | After CPET | 57/18 | 0.84 (0.56–1.26) | 0.395 | 56/29 | 0.70 (0.51–0.98) | 0.036 |
| NAC stage | Stage I | 54/10 | 0.64 (0.31–1.31) | 0.220 | 54/21 | 0.85 (0.56–1.28) | 0.436 |
| NAC stage | Stage II | 28/15 | 1.13 (0.67–1.91) | 0.639 | 28/21 | 0.88 (0.60–1.29) | 0.516 |
| NAC stage | Stage III | 0/0 | NE | NE | 1/0 | NE | NE |
| Diagnosis interval | Less than 1 year | 65/23 | 0.83 (0.56–1.24) | 0.365 | 67/32 | 0.76 (0.56–1.02) | 0.069 |
| Diagnosis interval | At least 1 year | 16/3 | NE | NE | 16/10 | 0.91 (0.57–1.46) | 0.703 |
| Restriction | Peak RER ≥1.05 | 83/24 | 0.62 (0.41–0.95) | 0.028 | 85/40 | 0.72 (0.54–0.96) | 0.027 |
| Restriction | Wild-type ATTR-CM | 96/28 | 0.62 (0.42–0.92) | 0.018 | 97/45 | 0.68 (0.52–0.90) | 0.006 |

All subgroup models adjust for age only. Sex remains in principal pooled models, but only seven women were included and several strata had no events among women. NE indicates fewer than five events or insufficient sample for estimation; this reporting floor does not ensure precision. The stage III group comprised one patient, with no valid mortality time and no composite event. Tafamidis strata reflect first recorded initiation dates, not confirmed ongoing exposure or never-treatment. Duration strata exclude 24 missing dates and one interval of −14 days. RER, respiratory exchange ratio.

# Supplementary Table S2 Additional adjustment in matched CPET samples

Each variable was added separately to the model containing age, sex, peak VO₂, VE/VCO₂ slope and HRR1. The comparison model used exactly the same patients. Estimates refer to HRR1 rather than to the added variable.

| **Outcome** | **Added variable** | **n/events** | **Matched CPET HR (95% CI)** | **P** | **CPET plus variable HR (95% CI)** | **P** |
| --- | --- | --- | --- | --- | --- | --- |
| Mortality | Recorded AF | 91/32 | 0.641 (0.433–0.951) | 0.027 | 0.645 (0.433–0.962) | 0.032 |
| Mortality | Beta-blocker | 103/33 | 0.646 (0.438–0.953) | 0.028 | 0.657 (0.449–0.962) | 0.031 |
| Mortality | Implanted rhythm device | 98/33 | 0.648 (0.441–0.952) | 0.027 | 0.646 (0.450–0.929) | 0.018 |
| Mortality | Calcium-channel blocker class | 102/33 | 0.648 (0.440–0.953) | 0.028 | 0.626 (0.416–0.944) | 0.025 |
| Mortality | Antiarrhythmic class | 102/33 | 0.648 (0.440–0.953) | 0.028 | 0.627 (0.425–0.927) | 0.019 |
| Mortality | Tafamidis initiation by CPET | 94/29 | 0.736 (0.496–1.091) | 0.127 | 0.740 (0.499–1.097) | 0.133 |
| Mortality | Diagnosis-to-CPET interval | 80/26 | 0.773 (0.509–1.172) | 0.225 | 0.771 (0.509–1.166) | 0.218 |
| Mortality | 24 W / peak workload | 104/33 | 0.646 (0.438–0.953) | 0.028 | 0.694 (0.480–1.004) | 0.053 |
| Composite | Recorded AF | 94/50 | 0.749 (0.577–0.970) | 0.029 | 0.740 (0.565–0.970) | 0.029 |
| Composite | Beta-blocker | 105/51 | 0.742 (0.570–0.967) | 0.027 | 0.742 (0.568–0.970) | 0.029 |
| Composite | Implanted rhythm device | 99/50 | 0.731 (0.560–0.954) | 0.021 | 0.740 (0.565–0.970) | 0.029 |
| Composite | Calcium-channel blocker class | 104/50 | 0.745 (0.570–0.974) | 0.031 | 0.754 (0.581–0.979) | 0.034 |
| Composite | Antiarrhythmic class | 104/50 | 0.745 (0.570–0.974) | 0.031 | 0.747 (0.571–0.979) | 0.034 |
| Composite | Tafamidis initiation by CPET | 94/47 | 0.803 (0.614–1.050) | 0.109 | 0.804 (0.616–1.051) | 0.110 |
| Composite | Diagnosis-to-CPET interval | 82/42 | 0.787 (0.595–1.041) | 0.093 | 0.794 (0.602–1.048) | 0.104 |
| Composite | 24 W / peak workload | 106/51 | 0.744 (0.571–0.970) | 0.029 | 0.765 (0.587–0.998) | 0.048 |

All covariates are baseline recorded characteristics. Drug class fields do not identify dose, amiodarone-specific use or calcium-channel blocker subtype. Tafamidis initiation is based on the first recorded start date; a later start identifies the comparator, and blank dates remain unknown. Diagnosis interval is continuous in years and non-negative. Relative recovery workload is 24 W divided by peak workload. Three decimals are shown to distinguish confidence bounds close to 1. These six-predictor models are exploratory and have limited events.

# Supplementary Table S3 Exploratory interaction tests

Each CPET-adjusted model included the subgroup variable and its interaction with continuous HRR1. Likelihood-ratio P-values compare the model with and without the interaction in identical complete-case samples.

| **Outcome** | **HRR1 interaction variable** | **n/events** | **Ratio of HRR1 hazard ratios (95% CI)** | **Interaction P** |
| --- | --- | --- | --- | --- |
| Mortality | Recorded AF | 91/32 | 1.693 (0.636–4.505) | 0.278 |
| Mortality | Beta-blocker | 103/33 | 1.582 (0.621–4.026) | 0.329 |
| Mortality | Implanted rhythm device | 98/33 | 0.453 (0.016–12.873) | 0.614 |
| Mortality | Tafamidis initiation by CPET | 94/29 | 0.469 (0.187–1.175) | 0.095 |
| Mortality | NAC stage II vs I | 82/25 | 1.778 (0.673–4.695) | 0.239 |
| Mortality | Diagnosis-to-CPET interval | 80/26 | 1.145 (0.766–1.709) | 0.527 |
| Composite | Recorded AF | 94/50 | 0.696 (0.415–1.167) | 0.174 |
| Composite | Beta-blocker | 105/51 | 1.699 (0.944–3.058) | 0.068 |
| Composite | Implanted rhythm device | 99/50 | 0.987 (0.456–2.133) | 0.973 |
| Composite | Tafamidis initiation by CPET | 94/47 | 1.075 (0.627–1.841) | 0.794 |
| Composite | NAC stage II vs I | 82/42 | 0.910 (0.500–1.655) | 0.756 |
| Composite | Diagnosis-to-CPET interval | 82/42 | 1.098 (0.867–1.390) | 0.441 |

Models adjust for age, sex, peak VO₂ and VE/VCO₂ slope. For binary variables, the ratio compares the HRR1 hazard ratio in the yes group with that in the no group. NAC testing compares stage II with stage I and excludes the single stage III patient. For diagnosis interval, the ratio corresponds to one additional year from diagnosis to CPET. These seven-predictor models have limited events; the device mortality interaction is particularly imprecise. Non-significant interactions do not demonstrate equality across strata. No multiplicity correction was applied.

# Supplementary Table S4 Matched biomarker available samples

Age- and sex-adjusted models with and without each biomarker were fitted to the same complete-case patients. Estimates refer to HRR1 per 10-bpm greater recovery.

| **Outcome** | **Available biomarker** | **n/events** | **Age and sex HR (95% CI)** | **P** | **Age sex and biomarker HR (95% CI)** | **P** |
| --- | --- | --- | --- | --- | --- | --- |
| Mortality | NT-proBNP | 91/26 | 0.72 (0.47–1.12) | 0.145 | 0.86 (0.55–1.34) | 0.509 |
| Mortality | NAC stage | 82/25 | 0.76 (0.49–1.18) | 0.222 | 0.86 (0.55–1.34) | 0.503 |
| Composite | NT-proBNP | 93/44 | 0.78 (0.59–1.03) | 0.078 | 0.86 (0.65–1.13) | 0.269 |
| Composite | NAC stage | 83/42 | 0.78 (0.59–1.04) | 0.089 | 0.82 (0.63–1.07) | 0.143 |

NT-proBNP is log₂-transformed; NAC stage is ordinal (1–3), as in the principal models. The smaller matched-sample HRR1 estimates were already non-significant before biomarker adjustment and moved towards unity afterwards. These data cannot distinguish the contributions of selection, precision and shared disease-severity information, and do not establish independence from biomarker staging.

# Supplementary Table S5 A Missing data in the HRR1 cohort

| **Variable** | **Available n** | **Missing or unusable n** | **Missing or unusable %** |
| --- | --- | --- | --- |
| Exact HRR1 | 110 | 0 | 0.0 |
| Age | 110 | 0 | 0.0 |
| Sex | 110 | 0 | 0.0 |
| Peak VO2 | 110 | 0 | 0.0 |
| VE/VCO2 slope | 109 | 1 | 0.9 |
| Recorded AF | 96 | 14 | 12.7 |
| Beta-blocker | 109 | 1 | 0.9 |
| Implanted rhythm device | 103 | 7 | 6.4 |
| Calcium-channel blocker class | 108 | 2 | 1.8 |
| Antiarrhythmic class | 108 | 2 | 1.8 |
| First tafamidis start date | 98 | 12 | 10.9 |
| NT-proBNP | 96 | 14 | 12.7 |
| NAC stage | 85 | 25 | 22.7 |
| Raw diagnosis-to-CPET interval | 86 | 24 | 21.8 |
| Non-negative diagnosis-to-CPET interval | 85 | 25 | 22.7 |
| Recovery-to-peak workload ratio | 110 | 0 | 0.0 |
| Valid mortality follow-up time | 105 | 5 | 4.5 |
| Valid composite follow-up time | 107 | 3 | 2.7 |

Denominator is 110 throughout. Follow-up counts denote valid times under the original outcome rules, rather than missing event indicators alone. One recorded diagnosis interval was −14 days and was excluded only from duration sensitivity analyses. Missing tafamidis dates remain unknown. No imputation was used.

Across AF, beta-blocker status, device status, tafamidis initiation, NT-proBNP, NAC stage, non-negative diagnosis interval and VE/VCO₂ slope, 62 patients had no missing variable. The most frequent missing patterns were NT-proBNP plus NAC stage (n=8), diagnosis interval alone (n=7), NAC stage alone (n=6) and NT-proBNP plus NAC stage plus diagnosis interval (n=3). These mutually exclusive patterns account for 86 patients; 18 other patterns account for the remaining 24. Full pattern counts are provided in Panel B below. The 62 complete patients are not a new primary cohort; the analyses use the complete cases required for each model.

# Supplementary Table S5 B Patterns of missing variables

Patterns are mutually exclusive across recorded AF, beta-blocker status, implanted rhythm device status, tafamidis initiation, NT-proBNP, NAC stage, non-negative diagnosis-to-CPET interval and VE/VCO₂ slope.

| **Missing or unusable variables** | **Patients** | **%** |
| --- | --- | --- |
| No missing variable | 62 | 56.4 |
| NT-proBNP + NAC stage | 8 | 7.3 |
| Diagnosis-to-CPET interval | 7 | 6.4 |
| NAC stage | 6 | 5.5 |
| NT-proBNP + NAC stage + Diagnosis-to-CPET interval | 3 | 2.7 |
| Recorded AF + Diagnosis-to-CPET interval | 2 | 1.8 |
| Recorded AF + NT-proBNP + NAC stage + Diagnosis-to-CPET interval | 2 | 1.8 |
| Recorded AF + Implanted rhythm device status + Tafamidis initiation + Diagnosis-to-CPET interval | 2 | 1.8 |
| Recorded AF + Tafamidis initiation + Diagnosis-to-CPET interval | 2 | 1.8 |
| Tafamidis initiation | 2 | 1.8 |
| Tafamidis initiation + Diagnosis-to-CPET interval | 2 | 1.8 |
| NAC stage + Diagnosis-to-CPET interval | 1 | 0.9 |
| NAC stage + VE/VCO₂ slope | 1 | 0.9 |
| Recorded AF | 1 | 0.9 |
| Recorded AF + Beta-blocker status + Implanted rhythm device status + Tafamidis initiation + Diagnosis-to-CPET interval | 1 | 0.9 |
| Recorded AF + NAC stage | 1 | 0.9 |
| Recorded AF + Implanted rhythm device status + Diagnosis-to-CPET interval | 1 | 0.9 |
| Recorded AF + Implanted rhythm device status + NAC stage + Diagnosis-to-CPET interval | 1 | 0.9 |
| Recorded AF + Tafamidis initiation | 1 | 0.9 |
| Implanted rhythm device status | 1 | 0.9 |
| Implanted rhythm device status + Diagnosis-to-CPET interval | 1 | 0.9 |
| Tafamidis initiation + NAC stage | 1 | 0.9 |
| Tafamidis initiation + NT-proBNP + NAC stage | 1 | 0.9 |

Denominator is 110 throughout. Diagnosis interval denotes the non-negative interval used in duration sensitivities; the −14-day interval is treated as unusable. No missing variable concerns these eight fields only and does not require complete follow-up. The 62 patients with all eight variables available do not replace the original primary cohort.

# Supplementary Table S6 Cohort selection and baseline comparison

Of 161 baseline patients, 11 were source-verification exclusions and 150 formed the data-quality cohort. The submitted exact-HRR1 cohort comprised 110 patients; the other 40 data-quality patients did not have eligible exact HRR1. The original selection is preserved.

| **Variable** | **Included HRR1 cohort (n=110)** | **Other data-quality patients (n=40)** | **Standardised difference** |
| --- | --- | --- | --- |
| Age (years) | 79 [75–83]; n=110 | 78 [72–82]; n=32 | 0.35 |
| Male sex | 103/110 (93.6%) | 31/40 (77.5%) | 0.47 |
| Peak VO2 (mL/kg/min) | 14.1 [11.6–17.6]; n=110 | 14.2 [11.3–17.8]; n=32 | 0.10 |
| VE/VCO2 slope | 38.4 [34.0–43.9]; n=109 | 36.0 [32.4–42.8]; n=31 | 0.38 |
| NT-proBNP (ng/L) | 2335 [1214–3527]; n=96 | 1981 [799–4710]; n=31 | -0.17 |
| Recorded AF | 51/96 (53.1%) | 16/34 (47.1%) | 0.12 |
| Beta-blocker | 63/109 (57.8%) | 22/40 (55.0%) | 0.06 |
| Implanted rhythm device | 15/103 (14.6%) | 4/36 (11.1%) | 0.10 |

Continuous variables are median [IQR] with available n; binary variables are count/available n (%). Standardised differences use means and pooled SDs for continuous variables and proportions for binary variables; signs indicate included minus unavailable. No assumption of random missingness is made. Missing baseline covariates within the 40 patients further limit the comparison.

# Supplementary Table S7 Recorded AF and HRR1 distributions

| **Recorded AF category** | **n** | **HRR1 bpm median [IQR]** |
| --- | --- | --- |
| No recorded AF | 45 | 15.0 [9.0–24.0] |
| Any recorded AF | 51 | 18.0 [9.0–30.0] |
| Permanent AF | 30 | 20.5 [12.2–32.8] |
| Paroxysmal AF | 14 | 12.0 [5.2–21.0] |
| AF type unspecified | 7 | 14.0 [3.0–25.0] |
| AF status unknown | 14 | 12.5 [8.2–18.5] |

The 51 patients with any recorded AF comprise 30 with permanent AF, 14 with paroxysmal AF and seven with unspecified AF type; these subtype rows are nested within the any-AF row. The remaining 45 patients had no recorded AF and 14 had unknown status. Any AF versus no AF: two-sided Mann–Whitney P=0.454. Recorded history does not establish rhythm during CPET.

# Supplementary Figure S1 Apparent calibration at three years

Apparent three-year calibration of the base CPET model (age, sex, peak VO₂ and VE/VCO₂ slope) and the otherwise identical model adding continuous HRR1. Panel A includes 104 patients with 33 deaths over follow-up; panel B includes 106 patients with 51 death or heart-failure hospitalisation events. At three years, 20 deaths and 39 composite events had occurred, with 49 and 45 patients remaining at risk, respectively. Each marker represents a quartile of model-predicted risk (26 patients per mortality quartile and 26–27 per composite quartile). The x-axis shows mean predicted risk and the y-axis shows the Kaplan–Meier event-risk estimate. Vertical bars show log–log Greenwood 95% confidence intervals. Intervals are omitted for quartiles with no observed events by three years; their zero-risk estimates are imprecise. The dashed diagonal indicates ideal calibration. These grouped estimates are descriptive and based on the model-fitting sample; bootstrap-corrected global calibration slopes and prediction error are reported in Table 3. CPET, cardiopulmonary exercise testing; HF, heart failure; HRR1, one-minute heart rate recovery.
